# Exploring the biological application of *Penicillium fimorum-*derived silver nanoparticles: *In vitro* physicochemical, antifungal, biofilm inhibitory, antioxidant, anticoagulant, and thrombolytic performance

**DOI:** 10.1016/j.heliyon.2023.e16853

**Published:** 2023-06-01

**Authors:** Hamed Barabadi, Kiana Mobaraki, Kamyar Jounaki, Salar Sadeghian-Abadi, Hossein Vahidi, Reza Jahani, Hesam Noqani, Omid Hosseini, Fatemeh Ashouri, Salimeh Amidi

**Affiliations:** a Department of Pharmaceutical Biotechnology, School of Pharmacy, Shahid Beheshti University of Medical Sciences, Tehran, Iran; b Department of Toxicology and Pharmacology, School of Pharmacy, Shahid Beheshti University of Medical Sciences, Tehran, Iran; c Department of Medicinal Chemistry, School of Pharmacy, Shahid Beheshti University of Medical Sciences, Tehran, Iran; d Shahid Beheshti University of Medical Sciences, Tehran, Iran

**Keywords:** Nano-biosynthesis, Mycosynthesis, Silver nanoparticles, Biological activity

## Abstract

This study showed the anti-candida, biofilm inhibitory, antioxidant, anticoagulant, and thrombolytic properties of biogenic silver nanoparticles (AgNPs) fabricated by using the supernatant of *Penicillium fimorum* (GenBank accession number OQ568180) isolated from soil. The biogenic AgNPs were characterized by using different analytical techniques. A sharp surface plasmon resonance (SPR) peak of the colloidal AgNPs at 429.5 nm in the UV–vis spectrum confirmed the fabrication of nanosized silver particles. The broth microdilution assay confirmed the anti-candida properties of AgNPs with a minimum inhibitory concentration (MIC) of 4 μg mL^−1^. In the next step, the protein and DNA leakage assays as well as reactive oxygen species (ROS) assay were performed to evaluate the possible anti-candida mechanisms of AgNPs representing an increase in the total protein and DNA of supernatant along with a climb-up in ROS levels in AgNPs-treated samples. Flow cytometry also confirmed a dose-dependent cell death in the AgNPs-treated samples. Further studies also confirmed the biofilm inhibitory performance of AgNPs against *Candia albicans*. The AgNPs at the concentrations of MIC and 4*MIC inhibited 79.68 ± 14.38% and 83.57 ± 3.41% of biofilm formation in *C. albicans*, respectively. Moreover, this study showed that the intrinsic pathway may play a significant role in the anticoagulant properties of AgNPs. In addition, the AgNPs at the concentration of 500 μg mL^−1^, represented 49.27%, and 73.96 ± 2.59% thrombolytic and DPPH radical scavenging potential, respectively. Promising biological performance of AgNPs suggests these nanomaterials as a good candidate for biomedical and pharmaceutical applications.

## 1 Introduction

Nanobiotechnology as a novel and promising branch of research deals with different aspects assessed in biotechnology and nanotechnology disciplines and represents biocompatible methods in various fields of science and industry. Preparation of materials in the nanoscale diameter, i.e., 1–100 nm, causes the emergence of unique chemical and physical properties that lack in the bulk material. This phenomenal potential is based on the morphology and size of the biofabricated nanomaterials. Hence, altering the procedure of the synthesis can lead to the formation of particles with different characteristics and therefore desirable properties [1]. Among different types of nanomaterials, metal-based nanoparticles (NPs) have has rightly attracted a great deal of attention owing to their unique physicochemical and biological properties [[2], [3], [4], [5], [6]].

Biosynthesis of NPs can be performed by employing different biological resources like bacteria, plants, algae, parasites, and fungi as reducing agents. NPs can be reduced either intracellularly or extracellularly that is based on the location of the NPs formation by biological metabolites and enzymes [7,8]. Bacterium-mediated synthesis of NPs is considered an environment-friendly and safe approach in which different species of bacteria are used to conduct the oxidation-reduction reaction. However, the bacteria used in this method may continue growing after the formation of NPs. Moreover, the variety of morphologies and diameters of the bacterium-assisted NPs is limited further confirming the need for other bioresources [9]. Plants as alternative biofactories provide a rapid method for biosynthesis of NPs with a high amount of reduction of the metallic ions [10]. Nevertheless, the metallic ions may have a harmful effect on the growth and metabolism of living plants [11].

Fungi on the other hand are considered the best-reducing agent among different microorganisms, as they are cost-effective, their growth is facile, the presence of mycelia induces the formation of a large surface-area ratio, and above all fungi can produce and secrete great amounts of proteins [12]. Hence, in the present study, fungus-assisted fabrication of AgNPs was examined using the fungal extract of *Penicillium fimorum* spp as the bioreductant. Although biological fabrication of NPs is the most beneficial method of synthesis with the least harm to nature, NPs can also be synthesized through physical and chemical routes. In a physical way, the bulk material breaks down into small pieces of NPs through the top-down approach in which various destructive tools like evaporation condensation ultra-sonication, electrochemical substances, microwave irradiation, and laser ablation are used [13]. Bottom-up technique as a more preferable approach deals with the transformation of basic substances like metallic ions to NPs employing chemical groups, sol-gel technique, spinning, and as discussed biological agents. Accordingly, sodium citrate, polyethylene glycol block copolymers, elemental hydrogen, N, *N*-dimethylformamide (DMF), sodium borohydride (NaBH_4_), tollens reagent, and ascorbate are among the chemical substances used in this approach [14]. It is worth mentioning that the physical and chemical methods have exhibited several drawbacks including the toxicity of the chemical residuals against the environment, the high cost attributed to the tools and substances needed for the process, and the complexity of the steps. Therefore, it is yet to replace other methods with the biological technique of synthesis [15].

One of the other remarkable application of nanobiotechnology is in the decrease of biofilm formed by bacterial strains. The extracellular polymeric substances (EPS) produced by bacteria result in the formation of a structural scaffold in which bacteria colonize and are not affected by antibiotics. This protective shield has damaged several medical devices and it is considered a threat to the proper function of many other cases. Hence, the antibiofilm potential of NPs is of high importance among different scholars [16]. Chandrasekharan and coworkers biofabricated AgNPs using logging residue from timber trees *Gmelina arborea* and assessed their efficacy against bacterial biofilms. The biofilm made by the pathogenic bacteria *Staphylococcus aureus*, *Pseudomonas aeruginosa, Bacillus cereus*, and *Escherichia coli* decreased considerably (24–62%) employing the green fabricated AgNPs with spherical shape and the sized ranged from 34 to 40 nm [17].

Additionally, different literature exists discussing the biosynthesis of AgNPs and their toxic effect on fungi. For instance, Al-Otibi et al. reported the biosynthesis of AgNPs using Cheeseweed mallow (*Malva parviflora* L.) with an average size of 50.6 nm and mostly spherical morphology. They examined the fungicidal activity of the biogenic AgNPs against *Alternaria alternate*, *Fusarium oxysporum*, *Fusarium solani*, and *Helminthosporium rostratum* of which *H. rostratum* demonstrated the maximum inhibition in mycelial growth of 88.6% [18]. Likewise, Jebril et al. performed an eco-friendly experiment *in vitro* and *in vivo* regarding the biofabricatoin of AgNPs and the examination of their antifungal property. Aqueous leaf extract of *Melia azedarach* was used as the reducing and stabilizing agent for the formation of AgNPs with spherical structure and diameter of 18–30 nm. It was confirmed that in the *in vitro* assay, the biologically prepared AgNPs at the concentration of 60 ppm could actively reduce the radial growth of *Verticillium dahliae* mycelia found in *Solanum melongena* L. (aubergine) compared to the control. Alternatively, the relative vascular discoloration and wilt severity of *V. dahliae* was significantly hindered by 97% and 87%, respectively using 20 ppm of the green synthesized AgNPs [19].

The thrombolytic property of biosynthesized NPs has also attracted great attention and different researchers have focused on the development of thrombolysis employing nanobiotechnology. Elegbede and coworkers reported promising results regarding the significant thrombolytic potential of the biological AgNPs. In this study, xylanases of *Trichoderma longibrachiatum* L2 (TEA) and *Aspergillus niger* L3 (NEA) were employed for the reduction of Ag ions to AgNPs with spherical shape and particle size ranging between 15.21 and 77.49 nm. It was noted that the thrombolytic activity of the NPs could be beneficial to drug delivery systems and coating processes as the contact of NPs with blood would not lead to coagulation [20]. In a similar study, Tag et al. biofabricated AgNPs using *Haloferax* sp. NRS1 and determined their characteristics by TEM analyses, which showed that the AgNPs possessed a diameter range of 5.77–73.14 nm and a spherical structure. The ecofriendly AgNPs displayed a concentration-dependent thrombolytic activity with the highest antithrombotic effect of 50.218% (*P* < 0.05) at the concentration of 100 μg mL^−1^ [21]. Another remarkable application of biological NPs is their role as antioxidant agents. Some of the studies investigated the formation of AgNPs and assessment of their antioxidant effect. Govindappa et al. reported the biosynthesis of AgNPs utilizing an aqueous extract of pomegranate fruit fleshy pericarp with a mostly spherical shape and an average diameter of 68 nm. It was indicated that the AgNPs exhibited great antioxidant activity, which was similar to ascorbic acid and butylated hydroxytoluene (BHT). It was also suggested that the presence of polyphenols in the fruit extract could improve the antioxidant effect of the phytofabricated AgNPs [22]. Accordingly, Genc et al. synthesized sphere-shaped AgNPs through an environment-friendly method. Further, the average size of the AgNPs was recorded as 54.2 nm according to SEM results. The antioxidant potential of the AgNPs was assessed through different techniques leading to promising results. The ABTS^•+^ scavenging effect of the NPs was significant with IC_50_ of 5.16 ± 0.07 μg mL^−1^ compared to standard BHT with IC_50_ of 7.03 ± 0.19 μg mL^−1^ [23].

Furthermore, NPs are considered suitable anticoagulant candidates in the future. In a study, Borah and coworkers investigated the ability of biologically synthesized AgNPs to inhibit the coagulation of blood. It was reported that the AgNPs prepared by utilizing dried biomass of a cyanobacterium, *Nostoc carneum* had a size ranging from 4 to 22 nm and were quasi-spherical morphologically. These biogenic AgNPs exhibited significant anticoagulant activity of more than 4 h [24]. Overall, the above examples showed a wide range of biological activities of biogenic AgNPs. Hence, the current study aimed to explore the *in vitro* physicochemical, antifungal, biofilm inhibitory, antioxidant, anticoagulant, and thrombolytic performance of *P. fimorum*-derived AgNPs. To the best of our knowledge, no earlier studies employed *P. fimorum* for green synthesis and characterization of AgNPs. Moreover, although the antimicrobial performance of mycosynthesized AgNPs was reported earlier, rare studies explored the anticoagulant and thrombolytic performance of mycosynthesized nanosized silver particles. An in-depth and step-by-step description of mycofabrication of AgNPs and their structural characteristics as well as their biological properties were discussed.

## 2 Materials and methods

### 2.1 Chemical materials and microbial strains

In this study, *Candida albicans* (ATCC 10231) were prepared from Microbiology Department, Central Research Laboratories, Shahid Beheshti University of Medical Sciences, Tehran, Iran. The compounds and chemical reagents used in different steps of the research were of analytical grade and obtained from Sigma-Aldrich, USA.

### 2.2 Isolation of *Penicillium fimorum* spp

The fungal strain was isolated from soil by coordination of 35.75453° N, 51.41907° E. The 10^−3^ diluted sample with phosphate-buffered saline (PBS) buffer (1:1000 dilution) was cultured on the fungal-specific solid medium of sabouraud dextrose agar (SDA) and incubated at 28 °C for 72 h. The purity of the isolated strain was confirmed by several subcultures and structural microscopic uniformity of mycelia after lactophenol cotton blue coloring.

### 2.3 Identification method

#### 2.3.1 Morphologic study

Initially, fungal colonies were cultured in the SDA medium for seven days. Macromorphological characteristics of the isolated strain were performed by using the lactophenol cotton blue staining method [25]. Lactophenol cotton blue (LCB) is a mixture of methyl blue, glycerol, phenol, and lactic acid and it is used for fungal structure visualization. LCB stains the chitin in the fungal cell walls blue. In the process of staining a piece of fungal mycelium was moved on the glass slide and submerged in 50 μL of LCB dye. Lamella was inserted with the pressure of the thumb and the slide was studied under the optical microscope.

#### 2.3.2 Genomic DNA extraction

The sequencing of ITS rDNA genes was carried out to identify the selected strain. Genomic DNA was extracted from fungal cells using a modified phenol-chloroform extraction method [26] to use as a template for PCR amplification. A_260_/A_280_ ratio was measured to verify the concentration and purity of template DNA.

#### 2.3.3 PCR

The ITS sequence was amplified using ITS1 (TCCGTAGGTGAACCTGCGG) and ITS4 (TCCTCCGCTTATTGATATGC) primers [27]. The PCR mixture (50 μL) contained 25 μL of Ampliqon™ Taq DNA Polymerase 2*x* Master Mix RED, 10 pM of each forward and reverse primer, and 50–100 ng of extracted genomic templet, according to the master mix guideline [28]. These reagents were combined in 200 μL micro-tubes and placed in a Primus 96 advanced thermal cycler programmed as 35 cycles, 94 °C for 45 s denaturation, 55 °C for 30 s primer annealing, and 72 °C for 60 s extensions.

#### 2.3.4 Sequencing and BLAST

The amplified DNA was separated on 1% agarose gel in TEA buffer. Besides, the purity of the PCR product was shown in the electrophoresis. The sequence of the PCR product was determined by Sanger method in Pishgam Co. (Tehran, Iran). Firstly, the sequence was submitted to the nucleotide bank of NCBI (OQ568180). Afterward, sequence comparisons were performed using BLAST (NCBI, USA). Phylogenetic and molecular evolutionary analyses were conducted using the molecular evolutionary genetics analysis (MEGA) software version 7.00. Distances and clustering were calculated using the neighbor-joining method. The fungus was maintained on SDA culture at 4 °C for long storage and sub-culturing was done every month.

### 2.4 Fungus-mediated synthesis of AgNPs

**Fig. 1:**
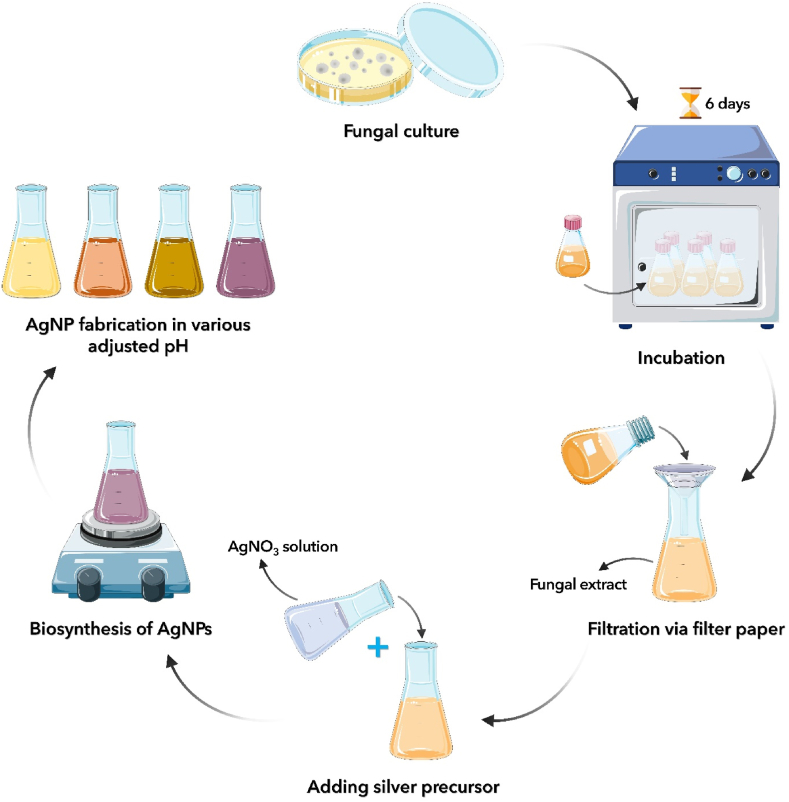
A schematic representation of the process mycosynthesis of AgNPs.

The fungus *P. fimorum* spp was cultured in Sabouraud Dextrose Broth (SDB) and incubated in a rotary shaker (JAL TAJHIZ®, JTSL 40, Iran) at 28 °C and 150 rpm for 6 days. In the next step, after centrifugation for 15 min at 8000 rpm, the supernatant was separated from the mycelia using a centrifuge (HETTICH®, ROTINA 380 R, Germany). Afterward, 100 mL of the supernatant was incorporated with 100 mL of AgNO_3_ solution (1 mM) followed by the preparation of different derivates having pH values of 8, 9, 10, and 11. Fig. 1 showed a schematic representation of the process of mycosynthesis of AgNPs. Accordingly, the mixture underwent incubation at 28 °C for 24 h and then was centrifuged for 20 min at 20,000 rpm by ultracentrifuge (Beckman, L90k, USA) to collect the biosynthesized AgNPs. Further, the purification step was carried out three times employing deionized double distilled water.

### 2.5 Physicochemical characterization of mycosynthesized AgNPs

#### 2.5.1 UV–visible spectroscopy

The formation of AgNPs was confirmed through two different techniques, one of which is the results obtained from UV–vis spectrophotometry. Moreover, the observation of color change in the reaction mixture from pale yellow to dark brown further displayed the presence of biofabricated AgNPs in the composition.

#### 2.5.2 Field emission scanning electron microscopy (FESEM) coupled with energy dispersive X-ray (SEM/EDX) spectroscopy

Characterization of the AgNPs by field emission scanning electron microscopy (FESEM) (Sigma VP, ZEISS, Germany) at 15 kV coupled with Energy-dispersive X-ray spectroscopy (EDX) analysis to study the morphology and elemental composition of biofabricated AgNPs, respectively.

#### 2.5.3 Dynamic light scattering (DLS)

A Zetasizer Nanoparticle Analyzer using Zetasizer 3600 with a scattering angle of 90° at 25 °C (Malvern, UK) was conducted to study the hydrodynamic diameter of the AgNPs.

#### 2.5.4 Fourier-transform infrared (FT-IR) spectroscopy

A 2-mm semitransparent disk was prepared by compressing the mixture of the AgNPs and KBr at the ratio of 1:100 for 2 min and then the disk was analyzed by FT-IR (Agilent, Cary 630 model, USA) at the wavelength of 400–4000 cm^−1^ for identification of the chemical groups present on the surfaces of the NPs.

#### 2.5.5 X-ray diffraction (XRD) spectroscopy

X-ray diffraction (XRD) spectroscopy (PANalytical X'Pert Pro X-Ray Diffractometer; X'Pert Pro MPD; Netherlands) was conducted to evaluate the crystalline nature of AgNPs using Cu Kα wavelength of 1.5406 Å operated at 30 kV voltage and a current of 100 mA at a 2θ angle pattern in the region of 20°–80°. Then, the obtained XRD pattern was compared with the Joint Committee on Powder Diffraction Standards (JCPDS) library to confirm the formation of AgNPs with crystalline structure.

#### 2.5.6 Thermogravimetric analysis (TGA)

Thermogravimetric analysis (TGA) of the mycosynthesized AgNPs was conducted in nitrogen atmosphere on a thermogravimetric analyzer (TGA50, Shimadzu Company, Japan). For that, 5 mg of AgNPs were placed in an aluminum pan (Shimadzu Company, Japan; Catalog number: 201–52943) of TGA and the sample was heated in a temperature range of 25–595 °C at a heat rate of 10 °C/min to determine the weight loss profile and the amount of fungal residue on the AgNPs through thermal degradation. The data was analyzed using the TA-60 software (Shimadzu Company, Japan).

### 2.6 Antifungal activity of mycosynthesized AgNPs

#### 2.6.1 Broth microdilution assay

##### 2.6.1.1 Evaluation of MIC

The lowest concentration of the compound by which the growth of the microbial strain can be actively hindered is known as MIC. In the present study, a 96-well microtiter was used for the evaluation of MIC, into twelve wells of which 100 μL SDB medium was poured. Then, the first well of the plate was seeded with 100 μL of the mycosynthesized AgNPs and the second well was seeded with 100 μL of the first well content. The biological AgNPs were seeded in wells number three to eleven in the same way and well number twelve remained unseeded and was considered the negative control. Afterward, 100 μL of *C. albicans* with a concentration of 10^6^ CFU/mL was transferred into each of the twelve wells. Amphotericin B was also evaluated as the standard antifungal agent using the same process. Eventually, after mixing the constituents, the plates were incubated for one day at 28 °C.

#### 2.6.2 Cell membrane integrity assays

##### 2.6.2.1 DNA leakage assay

After the preparation of a reference strain of *C. albicans* at the concentration of 0.5 McFarland which is equivalent to 10^8^ CFU/mL in the SDB, it was treated with AgNPs with the concentrations of MIC and 4*MIC. The next step was incubation for one day at 125 rpm and 28 °C followed by centrifugation for 5 min at 8000 rpm to separate the supernatant. At last, Thermo Scientific NanoDrop™ 2000 Spectrophotometer was employed for the evaluation of the optical density (OD) of the supernatant. The ratio of absorbance at 260 nm and 280 nm (A_260_/A_280_) was used to assess the purity of DNA.

##### 2.6.2.2 Protein leakage assay

Firstly, a reference strain of *C. albicans* at the concentration of 0.5 McFarland which is equivalent to 10^8^ CFU/mL was prepared in the SDB. After treating the sample with the biogenic AgNPs at the concentrations of MIC and 4*MIC, the sample was transferred into the incubator for one day at 125 rpm and 28 °C. Then, the supernatant was collected after centrifugation of the sample for 5 min at 8000 rpm and a 96-well microtiter plate (SPL Life Sciences, Korea) was poured with 25 μL of the supernatant using the bicinchoninic acid (BCA) Protein Quantification Kit (Pars Tous Company, Iran; Catalog Number: A101251). After adding 75 μL of the working reagent solution to the samples, they were put into the incubator for 1 h at 60 °C. Finally, a Hybrid Multimode Reader Cytation 3 (BioTek Company, USA) was utilized to measure the OD of the samples at 562 nm.

#### 2.6.3 ROS assay

Accumulation of ROS can be highly harmful to biological structures and affect intracellular and cellular functions, as they are unstable reactive molecules. For assessment of the level of ROS in cells, TPR-ROS Assay Kit (Teb Pazhouhan Razi, Iran) was used in which ROS sensitive probe 2,7-Dichlorodihydrofluorescein diacetate (DCFH-DA) was employed through a fluorometric assay. Accordingly, dichlorofluorescein (DCF) and the detection of fluorescence intensity at λ_em_ = 535 and λ_ex_ = 485 was based on the hydrolysis of DCFH-DA to DCFH which is a non-fluorescent compound by ester hydrolysis enzyme followed by rapid oxidation to strong green fluorescence DCF, the intensity of which is contributed to the amount of ROS in cells. In this study, after seeding a 96-well plate with *C. albicans* cells at a density of 1.5 × 10^8^/well for one day, the culture medium was refreshed and the cells were treated with fungal-mediated AgNPs for one day. Then, the procedure was performed according to the TPR kit protocol and at last, a Hybrid Multimode Reader Cytation 3 (BioTek Company, USA) was used to assess the fluorescence intensity.

#### 2.6.4 Flow cytometry-based quantitative assessment of cell viability

At first, a reference strain of *C. albicans* at the concentration of 0.5 McFarland, which is equal to 10^8^ CFU/mL, in the SDB broth was treated with biologically synthesized AgNPs at different concentrations. Incubation of the samples for one day at 125 rpm and 28 °C was carried out and in the next step, the supernatant was separated by centrifugation for 2 min at 3000 rpm and then discarded. Afterward, the plate was washed twice with the PBS buffer and then incubated with 2 μg mL^−1^ of propidium iodide (PI) dye for 20 min. After the assessment of the samples using a flow cytometer (BD FACSCalibur™, USA), FlowJo software (Tree Star, Inc., Ashland, OR, USA) was employed for analyzing the data. Notably, the standard antifungal drug amphotericin B was considered the positive control of the study.

### 2.7 Biofilm inhibitory potential of mycosynthesized AgNPs

The ability of biosynthesized AgNPs to inhibit biofilm formation in *C. albicans* was analyzed. For that, 200 μL of yeast isolate was seeded in each well of a 96-well microplate at the concentration of 10^8^ CFU/mL. The wells contained AgNPs or amphotericin B as the positive control, 2% (w/v) glucose, and SDB at the concentrations of MIC and 4*MIC. After incubation of the plate for one day at 28 °C, the content of the wells was discarded, and then the wells were seeded with 200 μL of sterile PBS (pH 7.4). Afterward, to remove the planktonic cells, the content of the wells was again discarded and this removal process was repeated thrice. Then, 200 μL of 95% ethanol was used for 15 min to fix the biofilm in the wells and once again, the content of the wells was discarded. After drying the wells, 200 μL of 1% crystal violet dye was seeded in each well and set for 5 min to stain the biofilm. The stain was then removed by washing the wells with 200 μL of sterile distilled water three times. Then, crystal violet dye from stained biofilm was solubilized using 200 μL of 33% glacial acetic acid. Eventually, a Hybrid Multimode Reader Cytation 3 (BioTek Company, USA) was utilized for the measurement of the OD of the solubilized dye at 570 nm. The intensity of the biofilm phenotypes was categorized as strong biofilm (4 × ODc < OD), moderate biofilm (2 × ODc < OD ≤ 4 × ODc), weak biofilm (ODc < OD ≤ 2 × ODc), and no biofilm (OD ≤ ODc). Notably, ODc stands for cutoff optical density which represents three standard deviations above the mean OD of the negative control. The percentage of biofilm inhibitory potential of the sample can also be calculated using the following formula:

Biofilm inhibitory effect (%) = [(OD_positive control_ − OD_sample_)/OD_positive control_] × 100

### 2.8 Antioxidant potential of mycosynthesized AgNPs

The 2, 2-diphenyl-1-picrylhydrazyl (DPPH) radical scavenging method was employed to assess the ability of biosynthesized AgNPs as antioxidant agent. Initially, 90% ethanol was used to prepare AgNPs solutions at the concentrations of 16, 100, and 500 μg mL^−1^ and a mixture of 1 mL of DPPH solution at the concentration of 0.3 mM and 2.5 mL of the AgNPs sample was prepared. After incubation of the mixture for 30 min at room temperature, an absorbance value at 518 nm was measured. Ascorbic acid at the concentration of 10 mg mL^−1^ was also regarded as a positive control. Notably, the test was performed thrice, and eventually, the percentage of the DPPH scavenging effect was measured through the following formula knowing that A_blank_, A_sample_, and A_control_ were considered the absorbance of the blank sample, sample, and control respectively:

DPPH scavenging effect (%) = [(A_control_ – (A_sample_– A_blank_)/(A_control_)] × 100

### 2.9 Anticoagulant activity of mycosynthesized AgNPs

After adding the blood sample of a healthy adult male volunteer to sodium citrate in a tube, the tube was centrifuged for 15 min at 1500 g to obtain the platelet-poor plasma (PPP). The next step was the evaluation of time (in seconds) needed for coagulation of the blood through performing the prothrombin time (PT) and activated partial thromboplastin time (aPTT) test using a coagulation analyzer (Coa DATA 501, LABitec, Germany). For PT assay, 45 μL of prewarmed PPP and 5 μL of biogenic AgNPs were incubated for 300 s at 37 °C. After transferring 100 μL prewarmed thromboplastin-D to the mixture, the time needed for the formation of blood clots was measured. On the other hand, in the aPTT test, 45 μL of prewarmed PPP, 50 μL of aPTT reagent, and 5 μL of the biological AgNPs were mixed and put into the incubator for 180 s at 37 °C. After adding 50 μL of 0.025 M prewarmed calcium chloride, the mixture was set to assess the time it takes for coagulation.

### 2.10 Thrombolytic activity of mycosynthesized AgNPs

The thrombolytic property of bioengineered AgNPs was assessed according to the protocol used by Devi and coworkers [29]. To do so, after weighing the empty Eppendorf tubes (W1), 500 μL blood was poured into the tubes and then transferred into the incubator at 37 °C for 30 min to form blood clots. Afterward, the weight of Eppendorf tubes containing the clots (W2) and consequently the weight of blood clots (W3) were measured. Then, 100 μL of the biogenic AgNPs at the concentrations of 1000 μg mL^−1^ and 500 μg mL^−1^ was added into the tubes containing blood clots. 100 μL of distilled water was also used as the negative control. As the next step, all of the tubes were incubated for 90 min at 37 °C and then the tubes were put upside down to analyze the lysis of blood clots. After discarding the lysed clots, the tubes containing the remaining clots were weighted (W4) to measure the weight of the remaining clots. Finally, the percentage of thrombolytic activity of the AgNPs was evaluated using the following formula:

$$Thrombolysis\;\left(\%\right)=\frac{W3-W5}{W3}\times 100$$

### 2.11 Statistical test

Graph Pad Prism software (San Diego, CA; version 7.0) was used for data analysis. To assess the differences among mean values and the comparison of the results obtained in this study, One-way Analysis of Variance (ANOVA) and Tukey post-test were applied. *P* < 0.05 was considered as statistically difference level. All experiments were repeated three times. All data were reported as the mean ± standard deviation (SD).

## 3 Results and discussion

### 3.1 Isolation and identification of *Penicillium fimorum* spp

#### 3.1.1 Morphological characterization of strain

**Fig. 2:**
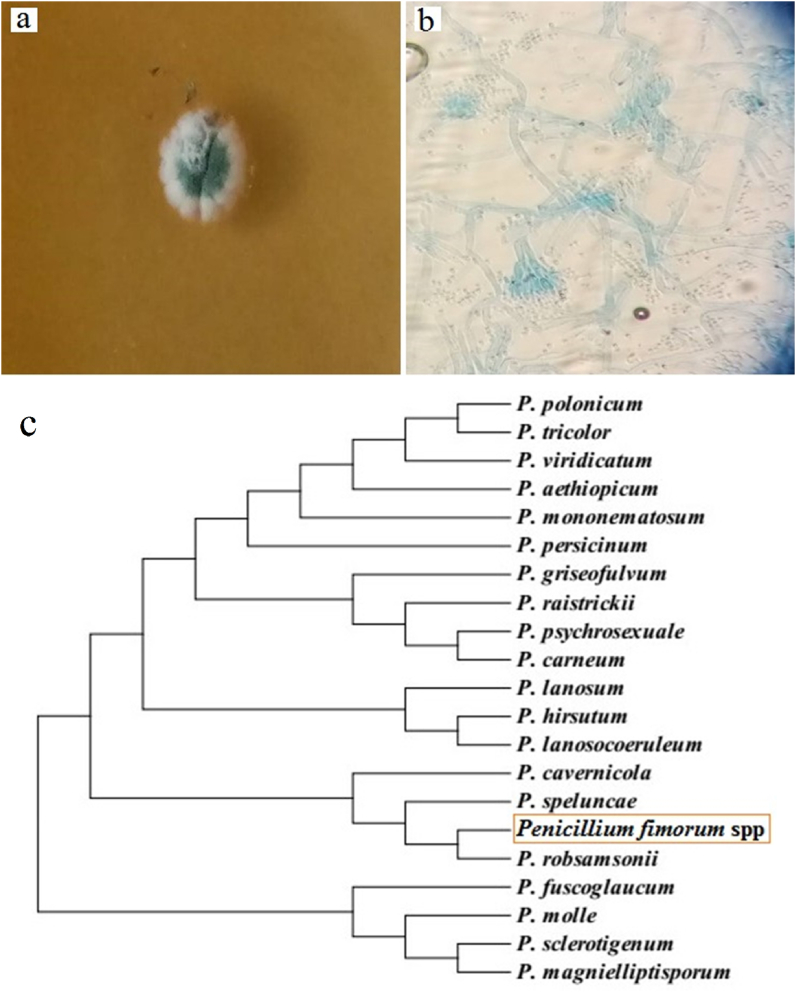
a) Colony morphology, and b) microscopic structure (400*X*) of the isolated strain; c) Phylogenic tree showing the inter-relationships of *P. fimorum* and its closest relatives based on the ITS sequences.

The isolated strain created fast-growing colonies, in shades of gray-green on SDA plates (Fig. 2a). The microscopic structure of the fungus was single-celled chains of mycelia ending in a brush-like appearance (a penicillus) of spores (Fig. 2b).

#### 3.1.2 ITS-based identification of strain

Identification studies based on ITS DNA sequencing revealed that this isolate most likely was (99.5% identity in the entire length of 605 bp with a maximum score of 1101) *Penicillum fimorum*. The sequence was also 99.3% identical to *P. robasamsonii* and 98.5% identical *to P. fuscoglaucum*. The sequence was deposited in NCBI GenBank under accession number OQ568180 and the phylogenetic tree was shown in Fig. 2c.

### 3.2 Mycosynthesis of AgNPs by employing *Penicillium fimorum* spp

The addition of silver nitrate to the fungal supernatant led to the appearance of dark brown color after 24 h confirming the bioreduction of silver ions and fabrication of nanosized silver particles. This color was darker for the sample with a pH value of 11. No color change was observed in the control in the same condition. The color change from pale yellow to dark brown following the fabrication of AgNPs was attributed to a phenomenon named surface plasmon resonance (SPR) resulting from the collective oscillation of free electrons of AgNPs in response to light in the visible region. These findings were in accordance with previous studies [[30], [31], [32]]. The exact mechanism of fungus-mediated synthesis of AgNPs has not been fully understood. However, a study reported that polypeptides had a key role in the *Penicillium cyclopium*-mediated fabrication of AgNPs [33]. In another study, a 2 kDa peptide containing 18 amino acids and the sequence was identified as GCSAAQGQGLCALKLSRL (Gly-Cys-Ser-Ala-Ala-Gln-Gly-Gln-Gly-LeuCys-Ala-Leu-Lys-Leu-Ser-Arg-Leu) produced by *Streptomyces clavuligerus* was identified as reducing and capping agent for the biofabrication of gold nanosized particles [34].

### 3.3 Physicochemical characterization of mycosynthesized AgNPs

#### 3.3.1 UV–visible spectroscopy

**Fig. 3:**
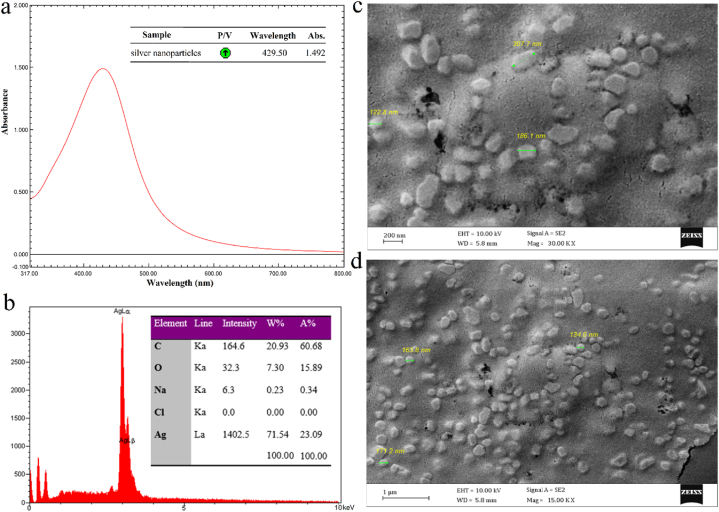
UV–vis absorbance spectrum (a), EDX spectrum (b), and FESEM micrographs of AgNPs with the scale bar of 200 nm (c) and 1 μm (d).

Fig. 3a showed a sharp SPR peak of the colloidal AgNPs with a pH value of 11 at 429.5 nm in the UV–vis spectrum confirming the fabrication of nanosized silver particles. No peak was seen in this area in 1 mM AgNO_3_ and fungal supernatant samples. The SPR peak of AgNPs usually occurs in the visible range of 380–450 nm in UV–vis spectrometry. Some parameters that influence the SPR peak of colloidal nanosized silver particles include particle size, morphology, and the interaction of particles with the medium like agglomeration [32].

#### 3.3.2 Energy-dispersive X-ray (EDX) spectroscopy

Fig. 3b showed the elemental composition of mycosynthesized AgNPs and represented a strong and sharp signal of silver atoms at an optical absorption band peak around 3 KeV. Other EDX signals were attributed to the X-ray emission of the elements in the structure of the biomolecules that were present in the supernatant and acted as reducing and capping/stabilizing agents. Similar findings were reported in previous studies [31,32,35].

#### 3.3.3 Scanning electron microscopy (SEM)

Fig. 3c–d showed the SEM micrographs of mycosynthesized AgNPs with different magnifications representing the fabrication of irregular and nearly spherical-shaped particles on a nanoscale with a smooth edges. Besides, the SEM images confirmed that the AgNPs were properly dispersed with no agglomerations.

#### 3.3.4 Dynamic light scattering (DLS) spectroscopy

**Fig. 4:**
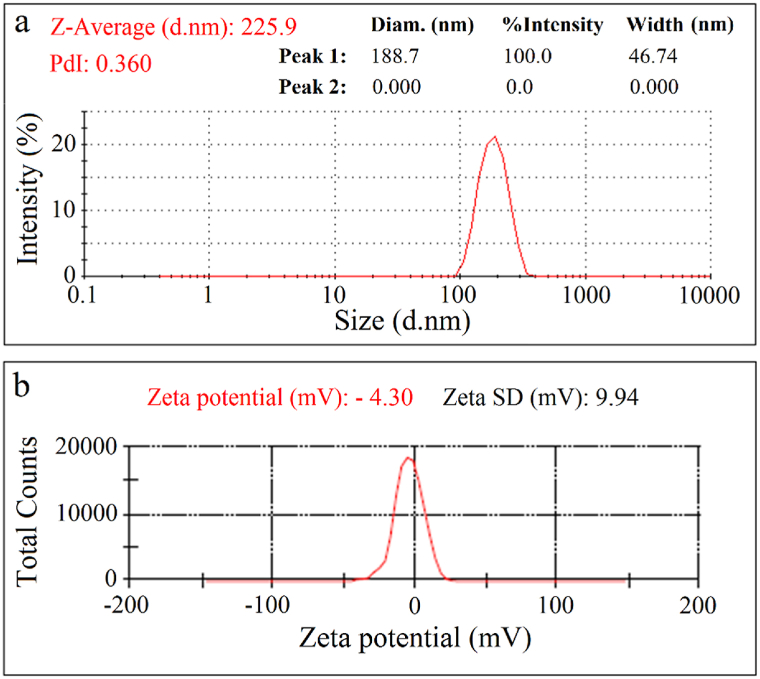
DLS spectrum (a) and zeta potential (b) of AgNPs.

Besides, as shown in Fig. 4a, the average hydrodynamic particle size of mycosynthesized AgNPs was found to be 225.9 nm with a polydispersity index (PdI) of 0.360. This data was obtained according to a technique named dynamic light scattering (DLS), also known as photon correlation spectroscopy (PCS). This technique is based on the Brownian motion of dispersed particles in a colloidal system. The hydrodynamic particle size of AgNPs is estimated by measuring the random changes in the intensity of light scattered from a colloidal system. Moreover, the PdI values above 0.7 correspond to the samples with a very broad particle size distribution [36]. In addition, a net negative charge (zeta potential) on the surface of mycosynthesized AgNPs was found to be −4 mV (Fig. 4b). The hydrodynamic particle size and surface charge are two key parameters that determine the characteristics of NPs for interaction with different macromolecules in biomedical sciences [37].

#### 3.3.5 Fourier-transform infrared (FT-IR) spectroscopy

**Fig. 5:**
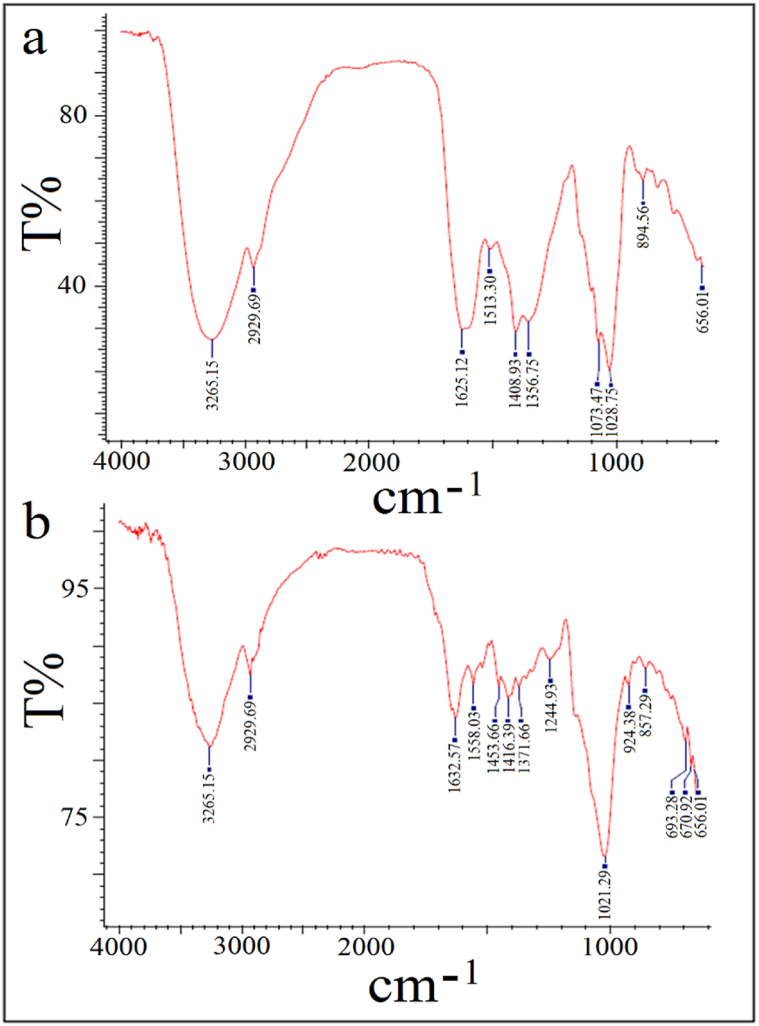
FT-IR spectrum of AgNPs (a) and *P. fimorum* pellets (b).

Furthermore, FT-IR spectral analysis was carried out to study the functional groups of the biomolecules adhered to the surface of AgNPs. Fig. 5a–b showed the FT-IR spectra of mycosynthesized AgNPs and *P. fimorum* spp pellets, respectively in the region of 450–4000 cm^−1^. Table S1 showed the peak and intensity list obtained from FT-IR spectroscopy of mycofabricated AgNPs and *P. fimorum* pellets. The FT-IR spectrum of AgNPs showed intensive peaks at 3265.15, 2929.69, 1625.12, 1513.30, 1408.93, 1356.75, 1073.47, 1028.75, 8944.56, and 656.01 cm^−1^ that may be assigned to the stretching vibration of *O*–H, *C*–H, *N*–H, C=C, *C*–C, *C*–H, *C*–N, *C*–N, *C*–H and *C*–H, respectively. In addition, the FT-IR spectrum of *P. fimorum* spp pellets exhibited absorption peaks at 3265.15, 2929.69, 1632.57, 1558.03, 1453.66, 1416.39, 1371.66, 1244.93, 1021.29, 924.38, 857.29, 693.28, 670.92, 656.01 cm^−1^ that may be assigned to the stretching vibration of *O*–H, *C*–H, *N*–H, C=C, *C*–H, *C*–C, *C*–H, *C*–N, *C*–N, *C*–H, *C*–H, *C*–H, *C*–H, and *C*–H, respectively. Although intensive HPLC/GCMS investigations are necessary to explore the exact biocomponents that play the role of reducing and stabilizing agents, the FT-IR revealed the presence of different functional groups in the sample. For example, the peak at 3265.15 cm^−1^ corresponds to *O*–H stretching of alcohols or phenols, the peak at 2929.69 cm^−1^ corresponds to alkane *C*–H stretching, the peaks at 1625.12 and 1632.57 cm^−1^ corresponds to *N*–H stretching of amines, the peak at 1408.93 cm^−1^ corresponding to *C*–C aromatic stretching, and the peak at 1356.75 cm^−1^ corresponding to *C*–H rocking vibration of alkanes [32]. Hence, the FT-IR results suggested the presence of phenolic compounds and proteins that were involved in the biosynthesis of AgNPs.

#### 3.3.6 X-ray diffraction (XRD) spectroscopy

**Fig. 6:**
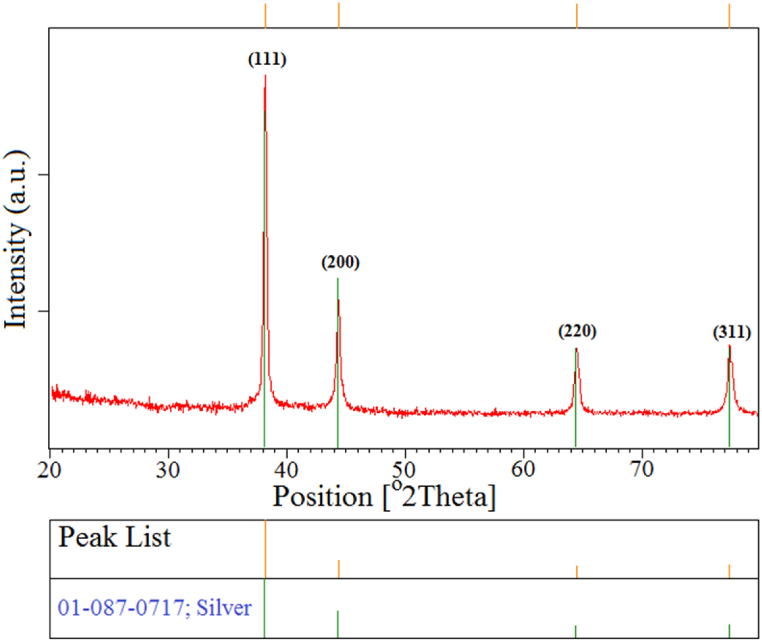
XRD spectrum representing the crystalline nature of biofabricated AgNPs.

The crystalline nature of biofabricated AgNPs was confirmed through an XRD pattern according to the standard JCPDS data (Ref. Code. 01-087-0717 (Ag)). As shown in Fig. 6, the XRD pattern exhibited the main peaks at (2θ) 38.11°, 44.30°, 64.45° and 77.40° that could be corresponded to the (111), (200), (220) and (311) planes, respectively which are characteristic of face centered cubic (FCC) structure of AgNPs. The XRD pattern showed no other impurities-related peaks representing the high purity of AgNPs. Our findings were in good agreement with previous studies [[38], [39], [40]].

#### 3.3.7 Thermogravimetric analysis (TGA)

**Fig. 7:**
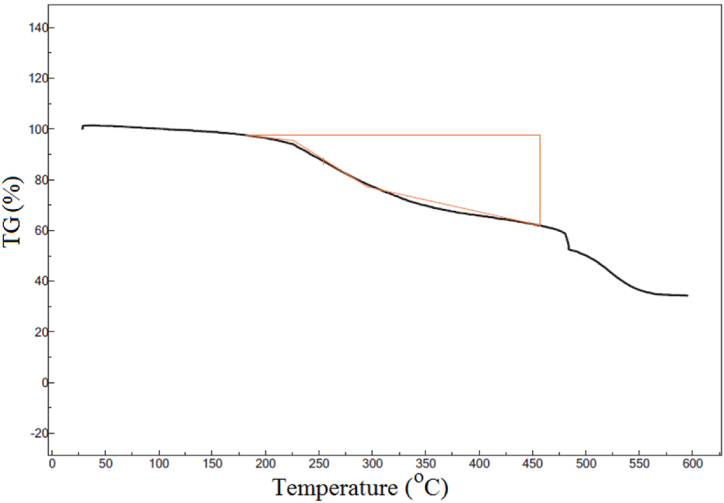
TGA spectrum of biofabricated AgNPs represented around 35.64% weight loss which could be attribute to the removal of bioorganic compounds adsorbed on the AgNPs surface.

The TGA was carried out to confirm the presence of fungal biomolecules as capping/stabilizing agents of the bioengineered colloidal AgNPs. Fig. 7 showed a temperature dependent weight loss profile. In detail, initial weight loss was observed in the temperature range of 150–200 °C because of the moisture loss. After 200 °C, the sample weight loss was observed with steeper slope up to 450 °C, which could be attribute to the removal of bioorganic compounds adsorbed on the AgNPs surface. As shown in Fig. 7, around 35.64% weight loss was observed resulting from thermal degradation of fungal bioorganic compounds. Our findings were in good agreement with previous studies [[41], [42], [43], [44]].

### 3.4 Antifungal activity of mycosynthesized AgNPs

#### 3.4.1 Broth microdilution assay

##### 3.4.1.1 Evaluation of MIC

**Fig. 8:**
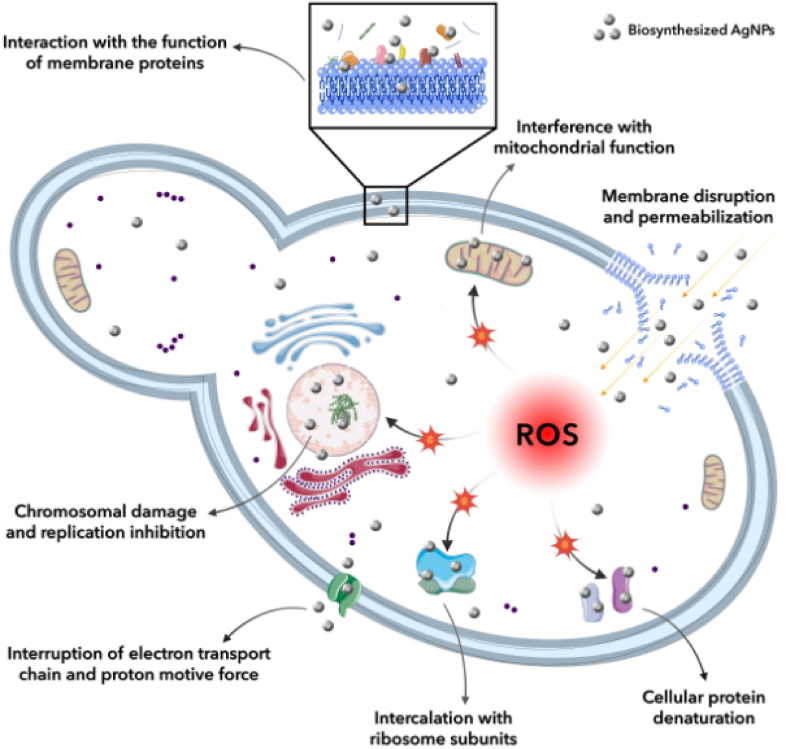
A schematic representation of possible antifungal properties of AgNPs.

The antifungal activity of mycosynthesized AgNPs was evaluated against reference *C. albicans* (ATCC 10231). Amphotericin B was selected as the positive control and the supernatant was the negative control of the study. The biogenic AgNPs and amphotericin B showed significant anti-yeast performance with MIC values of 4 and 0.5 μg mL^−1^, respectively, whereas the supernatant showed no anti-yeast activity against *C. albicans*. Previous studies also reported the anti-yeast properties of AgNPs. In a study, the spherical-shaped AgNPs were fabricated by employing cyanobacterium *Anabaena variabilis* in the range of 11–15 nm and showed considerable anti-yeast potential against pathogenic *C. albicans* and *C. glabrata* with MIC values of 12.5 and 25 μg mL^−1^, respectively [45]. Moreover, the polyvinylpyrrolidone capped-AgNPs showed a MIC value of ≤2 μg mL^−1^ against *C. albicans* (SC5314). According to the literature, the mycosynthesized AgNPs showed different performances against *C. albicans*. For example, the MIC value for *Aspergillus sydowii*-derived AgNPs was found to be 0.25 μg mL^−1^ against *C. albicans* (ATCC 90028) [46]. Besides, the MIC value for *Aspergillus terreus*-derived AgNPs was found to be 1.25 μg mL^−1^ against *C. albicans* (ATCC 10231) [32]. In addition, the MIC values for *Metarhizium roberts*-derived AgNPs were found to be 1.56 and 6.35 μg mL^−1^ against *C. albicans* (ATCC 10231) and *C. albicans* (ATCC 90028), respectively [47]. Furthermore, the MIC values for *Fusarium oxysporum*-derived AgNPs were found to be 1.68 μg mL^−1^ against both *C. albicans* (ATCC 10231) and *C. albicans* (ATCC 24433) [48]. The antifungal performance of AgNPs is highly dependent on their characteristics, such as particle size, morphology, surface coating, chemical composition, etc. Several mechanisms were reported for antifungal properties of biogenic AgNPs. Fig. 8 showed a schematic representation of proposed antifungal properties of AgNPs. Electrostatic attraction between AgNPs and surface of fungi results in the attachment of NPs to the surface of fungal cells with a further membrane damage and penetration of biogenic AgNPs inside the cells and induction of intracellular ROS. The overgeneration of ROS damages the intracellular organelles and triggers cell death [49]. Moreover, the silver cations can be released from AgNPs owing to the large surface area to volume ratio of AgNPs and these ions can bind to the enzyme protein sulfhydryl (–SH) and DNA bases and alter their functions [46]. Fouda et al. reported the biosynthesis of AgNPs using *Aspergillus flavus* F5 with an average diameter of 12.5 ± 5.1 nm and spherical morphology. The antifungal activity of the biological AgNPs was assessed against *Candida parapsilosis*, *C. tropicalis*, *C. glabrata*, and *C. albicans* with a MIC value of 12.5 ppm. AgNPs at the concentration of 100 ppm showed the highest inhibitory potential with inhibition zones of 15.4 ± 0.3, 15.8 ± 0.3, 17.8 ± 0.2, and 16.8 ± 0.3 mm corresponding to *C. parapsilosis*, *C. tropicalis*, *C. glabrata*, and *C. albicans*, respectively. The authors suggested different mechanisms underlying the fungicidal effect including cell apoptosis given the inhibition of bacterial signal transduction through dephosphorylation of tyrosine residue present on the peptide substrate, deactivation of respiratory enzymes, overproduction of ROS, accumulation of the NPs in the pits leading to the destruction of the cell membrane and cell wall, etc [50]. Similarly, Hawar et al. performed an experiment on forming AgNPs and evaluating their toxicity against *Candida* species. Leaf extract of *Alhagi graecorum* was used for the production of AgNPs with spherical shape and a diameter ranging from 22 to 36 nm. At 0.02 and 0.01 mmol/mL concentrations, the inhibition zones of 17–27 and 1–22 mm were recorded, respectively for *C. krusei*, *C. tropicales*, *C. parapsilosis*, *C. glabrata*, and *C. albicans*. The suggested mechanism by which the AgNPs hindered the growth of fungi was destroying the cell wall and interacting with sulfur and phosphorus groups and therefore binding to proteins and DNA [51]. Likewise, Elbahnasawy and colleagues synthesized AgNPs biologically utilizing *Rothia endophytica* with a cubic structure and size range of 47–72 nm. It was indicated that the green AgNPs had significant antifungal activity against *C. albicans* having MBC and MIC values of 125 and 62.5 μg/mL, respectively. The authors suggested that the penetration of the AgNPs through the cell wall leads to the deformation of its structure, increasing lipid peroxidation in the membrane, decreasing total proteins and sugars, and eventually leakage of the membrane-induced fungal cell impairment [52]. In a similar study, Singh et al. biofabricated spherical-shaped AgNPs using *Kinneretia* THGSQI4 with a size range of 15–20 nm. The green synthesized AgNPs were toxic to *C. albicans*, *C. tropicalis* with zones of inhibition of 20.5 and 15.5 mm, respectively. It was hypothesized that the dissipation of the proton motive force, the release of membrane proteins and lipopolysaccharides, the production of radicals, and the alternation of cell wall permeability were responsible for the antimicrobial activity of the AgNPs [53]. Abdallah and coworkers as well followed a biological approach for the synthesis of AgNPs with spherical morphology and a diameter ranging from 6 to 26 nm using the leaf extract of *Lotus lalambensis* Schweinf. AgNPs could actively hinder the growth of *C. albicans* with an inhibition zone of 24 mm. It was also noted that the antifungal activity was probably due to blockage of the cell cycle at the G2/M phase in the fungi, overgeneration of ROS, damaging the cell membrane, and reduction of antioxidant enzymes [54]. Alternatively, Yasir et al. phytofabricated AgNPs utilizing the leaf extract of *Syngonium podophyllum*. The green synthesized AgNPs were spherical morphologically and had an average size of 40 nm. Notably, the antifungal potential of 50 μg of the NPs against *C. albicans* was determined by evaluation of the maximum diameter of 15.60 mm as the inhibition zone. It was suggested that interaction of Ag ions with the pyrimidine and purine base pairs leading to the disruption of hydrogen binding and eventually denaturation of the DNA of the fungi. Moreover, accumulation of the ions inside the membrane and their penetration could destroy the cell membrane [55].

#### 3.4.2 Cell membrane integrity assays

##### 3.4.2.1 Protein leakage assay

**Fig. 9:**
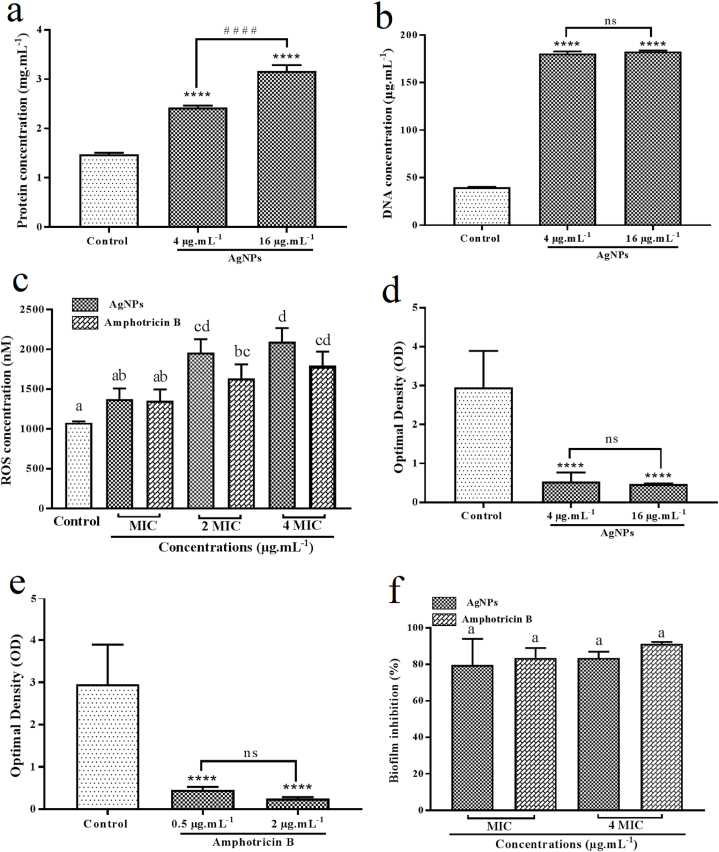
a) Total protein in the AgNPs-treated samples at the concentrations of 4 and 16 μg mL^−1^; b) The concentration of DNA in the AgNPs-treated samples at the concentrations of 4 and 16 μg mL^−1^; c) The ROS concentration in the treated samples with AgNPs and amphotericin B at the concentrations of MIC, 2*MIC and 4*MIC; d) The OD values in the AgNPs-treated samples at the concentrations of 4 and 16 μg mL^−1^; e) The OD values in the amphotericin B-treated samples at the concentrations of 0.5 and 2 μg mL^−1^; f) The percentage of inhibition of yeast biofilm for mycosynthesized AgNPs and amphotericin B at the concentrations of MIC and 4*MIC. Values are expressed as mean ± SD. **** refers to *P* < 0.0001 compared to the control group; ^# # # #^ refers to *P* < 0.0001 in two indicated groups; ns refers to not significant (*P* > 0.05); (n = 3) in each group. The difference between the groups that do not have a common letter is statistically significant (*P* < 0.05). AgNPs: silver nanoparticles; MIC: minimal inhibition concentration.

The BCA protein assay was used to quantify the amount of leakaged proteins from the damaged AgNPs-treated *C. albicans* cells into the medium. After 24 h of treatment of *C. albicans* cells with AgNPs at the concentrations of MIC and 4*MIC, a significant dose-dependent increase in the total protein of supernatant was found (*P* < 0.0001). The culture with no treatment was selected as the negative control of the study. As shown in Fig. 9a, the total protein in the AgNPs-treated samples at the concentrations of 4 and 16 μg mL^−1^ was found to be 2.42 ± 0.04 and 3.16 ± 0.12 mg mL^−1^, respectively. The negative control showed a total protein concentration of 1.47 ± 0.03 mg mL^−1^. It is noted that the difference between the samples and the negative control was statistically significant (*P* < 0.0001). Thereby, the results showed that the *C. albicans* cell membranes were disrupted by mycosynthesized AgNPs.

##### 3.4.2.2 DNA leakage assay

After 24 h of treatment of *C. albicans* cells with AgNPs at the concentrations of MIC and 4*MIC, the concentration of DNA in the supernatant was measured. The culture with no treatment was selected as the negative control of the study. As shown in Fig. 9b, the concentration of DNA in the AgNPs-treated samples at the concentrations of 4 and 16 μg mL^−1^ was found to be 180.60 ± 2.19 and 182.8 ± 0.92 μg mL^−1^, respectively. The negative control showed a DNA concentration of 40.07 ± 0.25 μg mL^−1^. It is noted that the difference between each sample and negative control was found to be statistically significant (*P* < 0.0001), whereas the difference between the AgNPs-treated samples with the concentrations of 4 and 16 μg mL^−1^ was found to be statistically insignificant (*P* > 0.05).

#### 3.4.3 ROS assay

After 24 h of treatment of *C. albicans* cells with AgNPs at the concentrations of MIC, 2*MIC, and 4*MIC, the ROS concentration in the supernatant was measured. Amphotericin B was selected as the positive control and the supernatant was the negative control of the study. As shown in Fig. 9c, the ROS concentration in the AgNPs-treated samples at the concentrations of 4, 8, and 16 μg mL^−1^ was found to be 1373 ± 135.69, 1960 ± 168.00 and 2099 ± 166.80 nM, respectively. Besides, the negative control showed a ROS concentration of 1079 ± 16.49 nM. Moreover, the ROS concentration in the amphotericin B-treated samples at the concentrations of 0.5, 1, and 2 μg mL^−1^ was found to be 1354 ± 142.50, 1636 ± 176.20 and 1796 ± 176.3 nM, respectively. No significant difference was found btween the ROS concentrations of the samples treated with the concentrations of MIC of AgNPs and amphotericin B compared to control (*P* > 0.05), whereas, significant difference was found btween the ROS concentrations of the samples treated with the concentrations of 2*MIC and 4*MIC of AgNPs and amphotericin B compared to control (*P* < 0.05).

#### 3.4.4 Flow cytometry-based quantitative assessment of cell viability

**Fig. 10:**
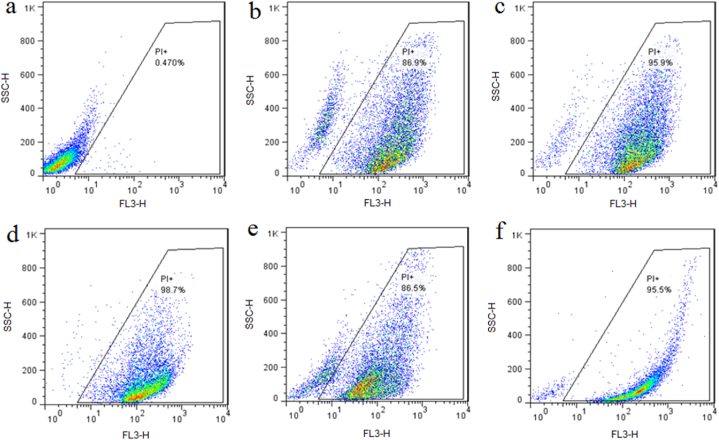
Flow cytometry-based quantitative assessment of *C. albicans* cell viability. a) Untreated *C. albicans* cells; b) AgNPs-treated cells at the concentration of 32 μg mL^−1^; c) AgNPs-treated cells at the concentration of 64 μg mL^−1^; d) AgNPs-treated cells at the concentration of 128 μg mL^−1^; e) Amphotericin B-treated cells at the concentrations of 1 μg mL^−1^; f) Amphotericin B-treated cells at the concentrations of 2 μg mL^−1^.

Flow cytometry was used to quantify the fungal viability of AgNPs-treated *C. albicans* cells in a mixed live-dead yeast population by employing PI dye to selectively stain dead cells. The red-fluorescent PI dye is a membrane-impermeant dye that can not pass into viable cells with intact membranes, but it can enter yeast cells with damaged membranes and bind to double-stranded DNA [56]. As shown in Fig. 10a, 99.53% of *C. albicans* cells were live in the untreated group. Besides, Fig. 10b, c and 10d showed 86.9%, 95.9%, and 98.7% yeast dead cells in the AgNPs-treated cells at the concentrations of 32, 64, and 128 μg mL^−1^, respectively. Moreover, Fig. 10e and f showed 86.5% and 95.5% yeast dead cells in the amphotericin B-treated cells at the concentrations of 1 and 2 μg mL^−1^, respectively.

### 3.5 Biofilm inhibitory potential of mycosynthesized AgNPs

**Fig. 11:**
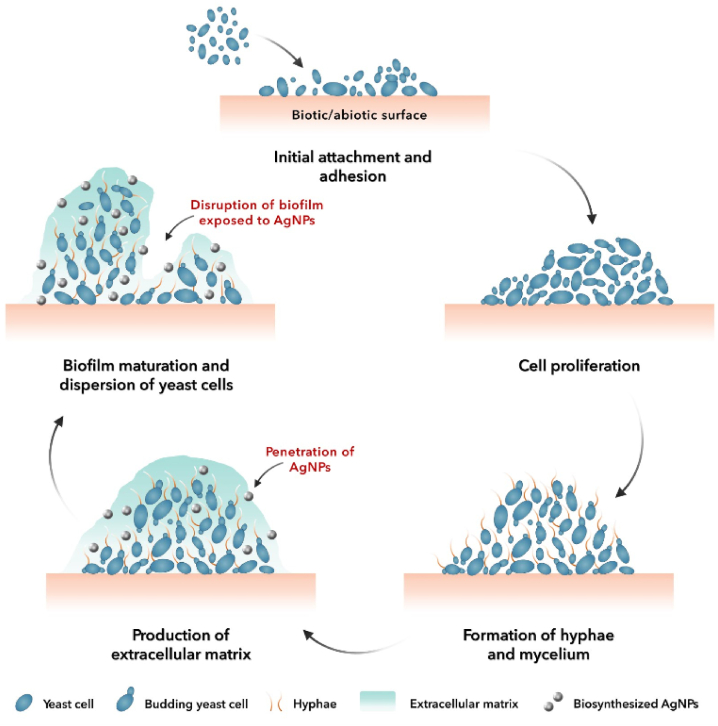
A schematic representation of the biofilm formation steps in *C. albicans* and the penetration of AgNPs into the biofilm leading to biofilm disruption.

The *C. albicance* biofilm inhibitory properties of mycosynthesized AgNPs and standard drug amphotericin B were tested at the concentrations of MIC and 4*MIC. To evaluate the yeast biofilm inhibitory performace of the mycosynthesized AgNPs, the average absorbance or mean OD reading from negative control wells, the ODc (optical density cutoff value), and the standard deviation (SD) of the negative control wells and the mean OD of growth control wells were found to be 0.2345, 0.280071, 0.01519, and 2.952, respectively. Fig. 9d showed the OD values in the AgNPs-treated samples at the concentrations of 4 and 16 μg mL^−1^ representing the significant inhibition of biofilm density in both treated samples compared to growth control (*P* < 0.0001). However, no significant difference was recorded in the biofilm inhibition effects of the AgNPs-treated samples at the concentrations of 4 and 16 μg mL^−1^ (*P* > 0.05). To compare the OD values of AgNPs-treated samples with growth control, the biofilm phenotype of weak appeared for both AgNPs-treated samples at the concentrations of MIC and 4*MIC. The evaluation of yeast biofilm inhibitory performance of the standard drug amphotericin B and mycosynthesized AgNPs was performed at the same time in one 96-well microtiter plate. Fig. 9e showed the OD values in the amphotericin B-treated samples at the concentrations of 0.5 and 2 μg mL^−1^ representing the significant inhibition of biofilm density in both treated samples compared to growth control (*P* < 0.0001). However, from the statistical point of view, no significant difference was recorded in the biofilm inhibition effects of the amphotericin B-treated samples at the concentrations of 0.5 and 2 μg mL^−1^ (*P* > 0.05). To compare the OD values of amphotericin B-treated samples with growth control, the biofilm phenotype of weak and negative appeared for amphotericin B-treated samples at the concentrations of MIC and 4*MIC, respectively. The percentage of inhibition of yeast biofilm for mycosynthesized AgNPs at the concentrations of MIC and 4*MIC was found to be 79.68 ± 14.38% and 83.57 ± 3.41%, respectively. Besides, the percentage of inhibition of yeast biofilm for amphotericin B at the concentrations of MIC and 4*MIC was found to be 83.62 ± 5.44% and 91.27 ± 1.02%, respectively. From the statistical point of view, no significant difference was recorded in the biofilm inhibition effects of AgNPs and amphotericin B at the concentrations of MIC and 4*MIC (*P* > 0.05) (Fig. 9f). Our findings exhibited considerable *C. albicans*-associated biofilm inhibitory effect of mycosynthesized AgNPs. *C. albicans* is an opportunistic yeast that can form biofilm and shelter the yeast cells for stable colonization to spread the pathogenesis of candidiasis. The formation of biofilm by *C. albicans* provides this opportunity for yeast cells to resist high doses of antifungal agents even at concentrations of 1000-fold higher than the planktonic yeasts IC_50_ [57]. The biofilm-inhibitory properties of AgNPs may be attributed to the inhibition of blastospores and hyphae. The development of yeast-to-hyphal transition facilitates the adhesion of *C. albicans* to the host surface resulting in biofilm formations that are necessary for the beginning of candidal pathogenesis. The AgNPs not only inhibit the formation of biofilm, but also penetrate the mature biofilm and disrupt the biofilm structure [58]. Fig. 11 showed a schematic representation of the biofilm formation steps in *C. albicans* and the penetration of AgNPs into the biofilm leading to biofilm disruption. Ahmad et al. performed an experiment on the assessment of biofilm inhibitory potential against *C. albicans* attributed to biogenic AgNPs. Cell extract of *Anabaena variabilis* was used for the synthesis of spherical AgNPs with a diameter ranging from 11 to 15 nm. The AgNPs at the concentration of 25 μg mL^−1^ induced 62.5% biofilm degradation and inhibition. Further, a MIC of 12.5 μg mL^−1^ was recorded for the biological AgNPs. The authors suggested that the biofilm inhibitory activity of AgNPs was probably due to shrinkage and morphological alternation of fungal cells, changes in permeability, and production of ROS [58]. Correspondingly, Alherz and colleagues biofabricated AgNPs utilizing leaf extract of *Encephalartos laurentianus* and determined their ability to reduce biofilm formation by *C. albicans*. The results obtained from characteristic techniques revealed that the NPs were spherical morphologically and had an average diameter of 18 ± 5 nm. In addition, MIC values of the green AgNPs ranged from 8 to 256 μg mL^−1^ against *C. albicans* and it was stated that biofilm formation was inhibited strongly and moderately, from 69.23% to 30.77%. Downregulation of the biofilm genes by AgNPs was found to be the probable mechanism responsible for the biofilm inhibitory effect. However, it was suggested that binding AgNPs to fungal cells, increasing penetration, and eventually, morphological changes played a role in the reduction of biofilm [59]. Similarly, Ansari et al. reported the green synthesis of AgNPs using leaf extract of *Terminalia catappa* with a mostly spherical structure and a size of 10.06 ± 0.84 nm in the mixture containing 1 mL of the herbal extract. It was indicated that the AgNPs at the concentration of 7.8 μg mL^−1^ were able to decrease the biofilm content formed by *C. albicans* by 63.63%. It was hypothesized that penetration and then the interaction of the biological NPs with key components that affected the biofilm formation like nucleic acids, proteins, lipids, and polysaccharides could lead to the inhibition of biofilm production [60]. Moreover, Al Aboody et al. biofabricated AgNPs utilizing latex of *Azadirachta indica* and investigated their effect on the inhibition of biofilm formed by *C. tropicalis*. According to SEM results, AgNPs had a diameter ranging from 17.4 to 40.9 nm, and different shapes of hexagonal, pentagonal, tetragonal, and triangular were observed. It was reported that the AgNPs at the concentrations of 4.12 and 3.25 μg mL^−1^ had biofilm inhibitory activity against fluconazole-resistant and fluconazole-susceptible *C. tropicalis* [61]. In a similar study, AgNPs were biosynthesized utilizing extracts of *Lycopersicon esculentum* with a spherical shape and size ranging between 10 and 50 nm. The *anti*-biofilm potential of the AgNPs was tested against *C. glabrata*, *C. parapsilosis*, and *C. albicans* and the results exhibited that *C. glabrata* and *C. albicans* were more susceptible to the AgNPs (MIC: 32 μg mL^−1^) comparing to *C. parapsilosis* (MIC: 8 μg mL^−1^). In addition, it was suggested that adherence of the AgNPs to the surface of the cells and then the formation of pores could affect the normal growth of the fungi [62]. Likewise, Muthamil et al. employed methanolic leaf extracts of *Hyptis suoveolens* and *Dodonaea viscosa* for the preparation of environment-friendly AgNPs with a spherical structure and size of 40–55 nm. The biofilm inhibitory activity of the AgNPs was significant against *Candida* species, i.e., *C. tropicalis*, *C. glabrata*, and *C. albicans*. Notably, it was demonstrated that more that 80% of the fungal biofilm was inhibited by the biogenic NPs at the concentration of 10 μg mL^−1^ [63].

### 3.6 Antioxidant potential of mycosynthesized AgNPs

**Fig. 12:**
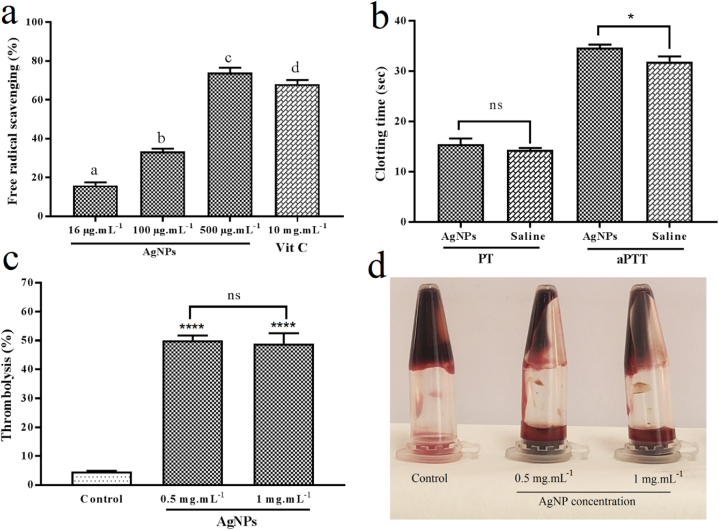
a) DPPH free radical scavenging of AgNPs at the concentrations of 16, 100, and 500 μg mL^−1^ compared to ascorbic acid; b) The coagulation screening tests (aPTT and PT values) of AgNPs at the concentrations of 0.5 mg mL^−1^ compared to saline; c) The percentage of thrombolytic activity of AgNPs at the concentrations of 0.5 and 1 mg mL^−1^ compared to distilled water; d) Visual observation of blood thrombolysis in the AgNPs-treated samples compared to distilled water. Values are expressed as mean ± SD. * and **** respectively refer to *P* < 0.05 and *P* < 0.0001 compared to the control group; ns refers to not significant (*P* > 0.05); (n = 3) in each group. The difference between the groups that do not have a common letter is statistically significant (*P* < 0.05). AgNPs: silver nanoparticles; aPTT: activated partial thromboplastin time; PT: prothrombin time.

DPPH free radical scavenging was used as an easy, quick, and affordable technique to measure the antioxidant properties of mycosynthesized AgNPs. The ascorbic acid was selected as the positive control of the study. As shown in Fig. 12a, the AgNPs at the concentrations of 16, 100, and 500 μg mL^−1^ showed a significant dose-dependent DPPH free radical scavenging of 15.96 ± 1.61%, 33.49 ± 1.41, and 73.96 ± 2.59%, respectively (*P* < 0.05). Besides, ascorbic acid at the concentration of 10 mg mL^−1^ showed 68.13 ± 2.06% antioxidant potential. Previous studies also reported the antioxidant properties of AgNPs. Khane and coworkers reported the biological fabrication of AgNPs with mainly spherical shapes and a diameter ranging from 7 to 28 nm utilizing aqueous *Citrus limon* zest extract. DPPH free radical scavenging test was performed for determination of antioxidant properties of the NPs and the best antioxidant activity was obtained at the IC_50_ of approximately 42.56 ± 0.02 μg mL^−1^. It was suggested that the antioxidant effect was attributed to the presence of functional groups (flavonoids and phenolic compounds) and their interaction with Ag ions having a synergistic effect on reducing the radicals [64]. Furthermore, Singh and colleagues biosynthesized AgNPs with face-centered cubic morphology and an average size of 35 ± 2 nm at 25 °C using leaf extract of *Carissa carandas* L. The results of DPPH free radical scavenging assay revealed that the best antioxidant activity of 90.3% was related to 15% AgNPs concentration at 25 °C. Further, the presence of flavonoids in the herbal extract coating the NPs was found to be effective in antioxidant capacity [65]. Likewise, Ansar et al. reported a green approach for the production of AgNPs with an average diameter of 20 nm and spherical structure utilizing *Brassica oleracea*. It was indicated that the biogenic NPs were able to scavenge free radicals effectively and the most antioxidant activity was recorded as 79% at the concentration of 200 μg mL^−1^ through a DPPH radical scavenging assay [66]. Alternatively, Bharathi and coworkers conducted a study on the biosynthesis of AgNPs and examination of their antioxidant potential. The NPs mediated by *Diospyros montana* stem bark extract were found to be mostly spherical with an average diameter of 28 nm and had dose-dependent antioxidant activity. By increasing the concentration of the AgNPs from 20 to 100 μg mL^−1^, more radicals were scavenged. It was believed that phytocompounds such as phenols present in the mixture could donate hydrogen atoms and played a significant role in reducing the free radicals [67]. Similarly, Baygar et al. utilized *Streptomyces griseorubens* AU2 as a reducing agent and fabricated spherical-shaped AgNPs biologically with a diameter ranging from 5 to 20 nm. It was indicated that AgNPs at concentrations of 20–100 μg mL^−1^ exhibited antioxidant activity of 9.66%–54.99% in a concentration-dependent manner [68]. In another study, AgNPs were produced through a green route using leaf extract of *Catharanthus roseus* with a mean size of 49 nm and spherical structure. The NPs showed the highest antioxidant effect of 82% at the concentration of 300 μg mL^−1^ and it was stated that the antioxidant activity was enhanced by increasing the concentration of the NPs. It is worth mentioning that phytochemicals present on the surface of the AgNPs contributed in their ability to scavenge free radicals [69].

### 3.7 Anticoagulant activity of mycosynthesized AgNPs

The aPTT and PT tests were performed to study the intrinsic and extrinsic pathways of blood coagulation, respectively. The anticoagulant activity of mycosynthesized AgNPs was evaluated at the concentration of 0.5 mg mL^−1^. As shown in Fig. 12b, the mean aPTT values for AgNPs and saline (negative control) were found to be 34.7 ± 0.06 and 31.93 ± 1.02 s, respectively. Besides, the mean PT values for AgNPs and saline were found to be 15.5 ± 1.15 and 14.4 ± 0.35 s, respectively. From the statistical point of view, the difference between the aPTT value of AgNPs and negative control was significant (*P* < 0.05), whereas the difference between the PT value of AgNPs and negative control was not significant (*P* > 0.05). These findings showed that the intrinsic pathway may play a significant role in the anticoagulant properties of mycosynthesized AgNPs. Aina et al. reported the biosynthesis of cubical AgNPs in the size range of 24.53–92.38 nm using the stem extract of *Chasmanthera dependens*. The anticoagulant activity of the NPs was determined by microscopic and visual observation and it was noted that 1 mM of the biological AgNPs could prevent the formation of clots in human blood for 1 h [70]. Likewise, Roy et al. used *Euphorbia canariensis* for the biofabrication of AgNPs with face-centered cubic shapes and a size range of 10–40 nm. It was observed that coagulation was prevented in the AgNP-treated human blood for 1 h [71]. In a similar paper, AgNPs were prepared by utilizing *Aspergillus niger*. The fungus-mediated AgNPs were spherical-shaped and had a diameter ranging from 15.21 to 77.49 nm. Visual and microscopic assays were performed for assessment of blood coagulation and it was concluded that no blood clots were detected for 4 h using AgNPs at the concentration of 100 μg mL^−1^ [72]. Alternatively, Talank et al. reported the green synthesis of AgNPs utilizing *Foeniculum vulgare* seeds with spherical structure and size ranging from 2 to 60 nm. The anticoagulant activity of the AgNPs was determined by measuring PT and aPTT values. Notably, PT recorded for AgNPs and normal saline as negative control were 15.43 ± 0.15 and 11.72 ± 0.29 s, respectively and aPTT values were 96.03 ± 1.65 and 29.98 ± 0.47 s corresponding to the biogenic AgNPs and normal saline. Accordingly, the rise in these values confirmed the effective anticoagulant potential of the NPs [73]. Azeez et al. as well studied the production of AgNPs biologically using *Coca beans*. The green AgNPs were in the size range of 8.96–54.22 nm and had spherical shapes. Microscopic and macroscopic observations revealed that 170 μg mL^−1^ of the AgNPs could inhibit the coagulation of human blood for 1 h [74]. In another study, the leaf extract of *Petiveria alliacea* was employed for the biological fabrication of nearly spherical AgNPs in a diameter ranging from 16.7 to 33.74 nm. The AgNPs were examined for anticoagulant potential and it was exhibited that human blood did not coagulate using AgNPs at the concentration of 100 μg mL^−1^ [75].

### 3.8 Thrombolytic activity of mycosynthesized AgNPs

The thrombolytic performance of mycosynthesized AgNPs was evaluated at the concentrations of 0.5 and 1 mg mL^−1^. Distilled water was selected as the negative control of the study. As shown in Fig. 12c, the percentage of thrombolysis was found to be 49.27%, 50.74%, and 4.78% for the AgNPs at the concentrations of 0.5 and 1 mg mL^−1^ and the distilled water, respectively. Besides, the lysis of the blood clot was shown in Fig. 12d. From the statistical point of view, the difference between the thrombolytic activity of AgNPs at the concentrations of 0.5 and 1 mg mL^−1^ was not significant (*P* > 0.05). According to the literature, two main mechanisms were suggested for the therombolytic activity of AgNPs involving a) direct breakdown of fibrin by AgNPs, and b) plasminogen activation with a further release of plasmin that dissolves the blood clot [76]. Previous studies also reported the thrombolytic properties of AgNPs. Nirubama et al. investigated the biosynthesis of AgNPs and evaluated their ability to lyse thrombi. The biogenic AgNPs were derived from *Andrographis echioides* and characterized as spherical with an average size of 10 nm. Applying 100 μL of AgNPs to 0.5 mL of human blood led to an average lysis of 50.7% after 24 h [77]. Similarly, Ananda et al. reported the phytofabrication of AgNPs with an average diameter of 79 nm and spherical morphology utilizing *Priva cordifolia*. The authors reported that 0.2 mL of the green AgNPs could immediately dissolve the blood clots [78]. Alternatively, Tag et al. used an eco-friendly route for the preparation of 27.7-sized AgNPs with mostly spherical shapes employing *Haloferax* sp. NRS. The thrombolytic activity of the AgNPs was confirmed by a decrease in the weight of blood clots. Accordingly, biogenic AgNPs at the concentration of 100 μg mL^−1^ showed an average of 50.218% blood clot lysis after 90 min. Notably, in all the doses tested the AgNPs were able to significantly lyse blood clots (*P* < 0.05) [21]. Correspondingly, Elegbede and coworkers employed *Trichoderma longibrachiatum* L2 for fungus-assisted synthesis of AgNPs. According to characteristic analyses, the biogenic AgNPs were spherical, cylindrical, and oval morphologically in the diameter ranging between 15.21 and 54.03. It was observed that the blood colts were dissolved by 170 μg mL^−1^ of the NPs after 5 min [72]. In a similar study, spherical-shaped AgNPs were produced using goat fur with a size ranging from 11 to 32 nm. Akintayo and coworkers indicated that 100 μL of the green fabricated AgNPs could actively induce 25% thrombolysis after 90 min [79]. Additionally, Lateef and colleagues reported the biological synthesis of AgNPs with nearly spherical structure and diameter of 5–40 nm using seed shell extract of *Cola nitida*. Notably, 100 μL of the green AgNPs exhibited great thrombolytic activity of 89.83% after 90 min [80].

## 4 Conclusion

The nanosized silver particles have attracted significant attention as a prominent nanomaterial for biomedical applications. The high surface-to-volume ratio provides unique physical, chemical, and optical properties for AgNPs to be widely utilized in various fields. In the current study, the supernatant of *P. fimorum* was successfully used for the extracellular biosynthesis of colloidal nanosized silver particles. The bioengineered AgNPs showed significant anti-yeast, biofilm inhibitory, anticoagulant, thrombolytic, and antioxidant performance. Promising biological performance of AgNPs suggests these nanomaterials as a good candidate for pharmaceutical and biomedical applications after careful evaluation of their acute and chronic safety. Thereby, further studies are required to evaluate the safety profile of these nanosized particles as well as their pharmacokinetics and pharmacodynamics in the body.

## Author contribution statement

Hamed Barabadi: Conceived and designed the experiments; Performed the experiments; Analyzed and interpreted the data; Contributed reagents, materials, analysis tools or data; Wrote the paper.

Kiana Mobaraki; Kamyar Jounaki; Salar Sadeghian-Abadi,; Hesam Noqani; Fatemeh Ashouri: Performed the experiments; Wrote the paper.

Hossein Vahidi; Omid Hosseini: Analyzed and interpreted the data; Contributed reagents, materials, analysis tools or data.

Reza Jahani; Salimeh Amidi: Conceived and designed the experiments; Analyzed and interpreted the data; Contributed reagents, materials, analysis tools or data.

## Data availability statement

Data included in article/supp. Material/referenced in article.

## Declaration of competing interest

The authors declare that they have no known competing financial interests or personal relationships that could have appeared to influence the work reported in this paper.
