# Supplementary material for: Exploring the biological application of Penicillium fimorum-derived silver nanoparticles: In vitro physicochemical, antifungal, biofilm inhibitory, antioxidant, anticoagulant, and thrombolytic performance
Source: Heliyon. 2023 Jun 1;9(6):e16853. doi: 10.1016/j.heliyon.2023.e16853 (PMC10258451; doi:10.1016/j.heliyon.2023.e16853)
Supplement: Multimedia component 1 [file mmc1.docx]

**Table S1.** The peak and intensity list obtained from FT-IR spectroscopy of mycofabricated AgNPs and *P. fimorum* pellets.

| **AgNPs** | | ***P. fimorum* pellets** | |
| --- | --- | --- | --- |
| **Peak** | **Intensity** | **Peak** | **Intensity** |
| 656.01 | 50.36 | 656.01 | 70.23 |
| 894.56 | 27.31 | 670.92 | 68.90 |
| 1028.75 | 100.00 | 693.28 | 60.82 |
| 1073.47 | 82.78 | 857.29 | 38.25 |
| 1356.75 | 72.21 | 924.38 | 43.27 |
| 1408.93 | 77.23 | 1021.29 | 100.00 |
| 1513.30 | 44.99 | 1244.93 | 35.42 |
| 1625.12 | 75.96 | 1371.66 | 43.72 |
| 2929.69 | 51.00 | 1416.39 | 47.31 |
| 3265.15 | 81.21 | 1453.66 | 43.69 |
|  |  | 1558.03 | 43.37 |
|  |  | 1632.57 | 53.30 |
|  |  | 2929.69 | 40.14 |
|  |  | 3265.15 | 62.49 |
