# Supplementary material for: Exploring the biological application of Penicillium fimorum-derived silver nanoparticles: In vitro physicochemical, antifungal, biofilm inhibitory, antioxidant, anticoagulant, and thrombolytic performance
Source: Heliyon. 2023 Jun 1;9(6):e16853. doi: 10.1016/j.heliyon.2023.e16853 (PMC10258451; doi:10.1016/j.heliyon.2023.e16853)
Supplement: Multimedia component 2 [file mmc2.pdf]

Form to confirm authorship changes for *Heliyon*

This form must be ***signed by all authors*** when there is a change in authorship which includes changes to any of the following items: author name(s), order of the authors, the corresponding author(s), the addition of authors, the removal of authors and changes in affiliation.

By personally signing this note, **all** authors confirm that: I) the changes are in accordance with their scientific contribution, II) they agree with all the changes and III) confirm that the authorship list conforms to the authorship criteria outlined on *Heliyon's* ethics page. IV) it is the responsibility of the corresponding author to get the signature from all co-authors accepting the change. In case of any ethic violation/malpractice in the signature, the corresponding author is accountable. The completed form should be returned along with the final/revised manuscript to proceed further with the manuscript. Manuscripts for which incomplete forms have been submitted will be rejected within 5 working days.

Any disputes on the authorship list and contributions need to be resolved by the involved scientists and *Heliyon* will only proceed with the evaluation of the manuscript once we receive confirmation, through this form, that such an agreement between the authors has been reached.

Manuscript number: HELIYON-D-23-09425R1

Article title: Exploring the biological application of Penicillium fimorum-derived silver nanoparticles: In vitro physicochemical, antifungal, biofilm inhibitory, antioxidant, anticoagulant, and thrombolytic performance

Complete new author list: Hamed Barabadi, Kiana Mobaraki, Kamyar Jounaki, Salar Sadeghian-Abadi, Hossein Vahidi, Reza Jahani, Hesam Noqani, Omid Hosseini, Fatemeh Ashouri, Salimeh Amidi

Date: 28th May 2023

#	First name	Last name	Order change (Y/N)	Addition / Deletion	Change in Author name (Y/N)	Affiliation Change (Y/N)	Reason for the change	Signature
1	Hamed	Barabadi	N	N	N	N		Barabadi
2	Kiana	Mobaraki	N	N	N	N		Kiana Mobaraki
3	Kamyar	Jounaki	Y	N	N	N	He significantly contributed to revise and modify the manuscript and answer the reviewers' questions.	
4	Salar	Sadeghian-Abadi	Y	N	N	N	He significantly contributed to revise and modify the manuscript and answer the reviewers' questions.	S. Sadeghian
5	Hossein	Vahidi	Y	N	N	N		HOSSEIN VAHIDI
6	Reza	Jahani	Y	N	N	N		Jahani
7	Hesam	Noqani	N	Y	N	N	This person was included because the reviewers requested new analytical experiments, such as TGA and XRD. The new author was involved to perform new analytical experiments.	Noqani
8	Omid	Hosseini	Y	N	N	N		O. Hosseini
9	Fatemeh	Ashouri	Y	N	N	N		F. Ashouri
10	Salimeh	Amidi	Y	N	N	N		Amidi Salimeh Amidi
